# The Mitochondrial Genomes of the Zoonotic Canine Filarial Parasites *Dirofilaria* (*Nochtiella*) *repens* and *Candidatus* Dirofilaria (Nochtiella) Honkongensis Provide Evidence for Presence of Cryptic Species

**DOI:** 10.1371/journal.pntd.0005028

**Published:** 2016-10-11

**Authors:** Esra Yilmaz, Moritz Fritzenwanker, Nikola Pantchev, Mathias Lendner, Sirichit Wongkamchai, Domenico Otranto, Inge Kroidl, Martin Dennebaum, Thanh Hoa Le, Tran Anh Le, Sabrina Ramünke, Roland Schaper, Georg von Samson-Himmelstjerna, Sven Poppert, Jürgen Krücken

**Affiliations:** 1 Institute for Parasitology and Tropical Veterinary Medicine, Freie Universität Berlin, Berlin, Germany; 2 Institute of Medical Microbiology, Justus-Liebig-University, Giessen, Germany; German Center for Infection Research (DZIF), Partner site Giessen-Marburg-Langen, Campus Giessen, Giessen, Germany; 3 IDEXX Laboratories, Ludwigsburg, Germany; 4 Institut für Parasitologie, Universität Leipzig, Leipzig, Germany; 5 Department of Parasitology, Faculty of Medicine Siriraj Hospital, Mahidol University, Bangkok, Thailand; 6 Department of Veterinary Medicine, University of Bari, Bari, Italy; 7 Division of Infectious Diseases and Tropical Medicine, Medical Centre of the University of Munich (LMU); German Center for Infection Research (DZIF), Partner site Munich, Germany; 8 Section Clinical Tropical Medicine, Department of Infectious Diseases, Heidelberg University Hospital, Heidelberg, Germany; 9 Immunology Department, Institute of Biotechnology, Ha Noi, Viet Nam; 10 Department of Parasitology, Viet Nam Veterinary Medical University, Ha Noi, Viet Nam; 11 Bayer Animal Health GmbH, Monheim, Germany; 12 University Medical Center, Hamburg-Eppendorf, Germany; University of Liverpool, UNITED KINGDOM

## Abstract

**Background:**

Cutaneous dirofilariosis is a canine mosquito-borne zoonosis that can cause larva migrans disease in humans. *Dirofilaria repens* is considered an emerging pathogen occurring with high prevalence in Mediterranean areas and many parts of tropical Asia. In Hong Kong, a second species, *Candidatus* Dirofilaria hongkongensis, has been reported. The present study aimed to compare mitochondrial genomes from these parasites and to obtain population genetic information.

**Methods and Findings:**

Complete mitochondrial genomes were obtained by PCR and Sanger sequencing or ILLUMINA sequencing for four worms. Cytochrome oxidase subunit 1 sequences identified three as *D*. *repens* (all from Europe) and one as *C*. D. hongkongensis (from India). Mitochondrial genomes have the same organization as in other spirurid nematodes but a higher preference for thymine in the coding strand. Phylogenetic analysis was in contradiction to current taxonomy of the Onchocercidae but in agreement with a recent multi-locus phylogenetic analysis using both mitochondrial and nuclear markers. *D*. *repens* and *C*. D. hongkongensis sequences clustered together and were the common sister group to *Dirofilaria immitis*. Analysis of a 2.5 kb mitochondrial genome fragment from macrofilaria or canine blood samples from Europe (42), Thailand (2), India (1) and Vietnam (1) revealed only small genetic differences in the *D*. *repens* samples including all European and the Vietnam sample. The Indian *C*. D. hongkongensis and the two Thai samples formed separate clusters and differences were comparatively large.

**Conclusion:**

Genetic differences between *Dirofilaria* spp. causing cutaneous disease can be considerable whereas *D*. *repens* itself was genetically quite homogenous. *C*. D. hongkongensis was identified for the first time from the Indian subcontinent. The full mitochondrial genome sequence strengthens the hypothesis that it represents an independent species and the Thai samples might represent another cryptic species, *Candidatus* Dirofilaria sp. ‘Thailand II’, or a quite divergent population of *C*. D. hongkongensis.

## Author Summary

The mitochondrial genomes of the zoonotic parasite species *Dirofilaria repens* and *Candidatus* Dirofilaria hongkongensis were characterized and compared to the genomes of other filariae. The resulting phylogeny is largely in agreement with recent molecular data. *C*. D. hongkongensis was placed as a sister group to *D*. *repens* and both as a common sister to *D*. *immitis*. The large genetic difference between *D*. *repens* and *C*. D. hongkongensis further supports the hypothesis that both are distinct valid species. Two canine samples from Thailand that were diagnosed as *D*. *repens* are either from a *C*. D. hongkongensis population that is quite divergent from the Indian population or might represent another currently unrecognized species in the genus.

## Introduction

The genus *Dirofilaria* contains several filarial nematode species that use various carnivores as definitive hosts. The species *Dirofilaria immitis* and *Dirofilaria repens* are clearly considered to be the most frequently found members of the genus and both infect predominantly canids [1–3]. While *D*. *immitis* is distributed in the Americas, Mediterranean area, many parts of Asia and Africa as well as Australia, *D*. *repens* is limited to the old world with endemic areas in Southern and Eastern Europe, many parts of Asia and presumably Africa, although there is only sparse information for the latter continent [4]. A new *Dirofilaria* species, *Candidatus* Dirofilaria hongkongensis has been proposed as a causative agent of subcutaneous or subconjunctival dirofilariosis in humans in Hong Kong and was also detected in dogs [5, 6].

Although *Dirofilaria* parasites can be found in many temperate climatic zones, transmission intensities are predicted to be much higher in tropical climate as revealed by modeling the extrinsic development in mosquitoes using climate data from South America [7]. Using logistic regression analysis, Wang et al. [8] showed that the odds of *D*. *immitis* prevalence increased significantly with increased temperature and precipitation in the USA, which also suggests that the parasites will be found particularly often in tropical climate zones. This is further corroborated by the fact that most of the known *Dirofilaria* species described currently have only been reported from countries with tropical climate [9, 10].

In humans as accidental hosts, *Dirofilaria* spp. usually do not complete their entire life cycle, although a few cases of infections with circulating microfilaria (MF) have been reported [11, 12]. Pulmonary dirofilariosis appears to be the most common clinical presentation in humans infected with *D*. *immitis*. In contrast, most cases of human infections with *D*. *repens* larvae or preadults are found in skin nodules and conjunctiva, but can also occur in inner organs such as lungs and testicles [13, 14]. In the worst cases, involving patent infections with production of MF, even functions of the central nervous system can be impaired with signs of meningoencephalitis, aphasia and apraxia [15]. In the absence of circulating MF and reliable serological tests, diagnosis of human dirofilariosis is usually limited to obvious cases such as those involving skin nodules or ocular complications or to the “accidental” detection of associated lesions in radiological images [16]. The latter are often initially considered to be malignancies that are surgically removed to obtain a definitive diagnosis resulting in worries and high costs [17]. Therefore, it is not surprising that human dirofilariosis is considered to be underdiagnosed [18]. Most likely, this is in particular true for tropical areas where people have only limited access to health facilities and advanced imaging methods and where biopsies for histological diagnosis are usually not feasible. In Europe, where *D*. *immitis* and *D*. *repens* are co-endemic, the vast majority of human cases can apparently be attributed to *D*. *repens* [11, 19] although recently two confirmed human *D*. *immitis* cases have been reported from Italy [20, 21]. In contrast, *D*. *immitis* is well known to be the causative agent of many cases of human dirofilariosis in North America [10] and Japan [22, 23] where *D*. *repens* is not endemic. The different frequency of human infections suggest that *D*. *immitis* is in fact a cryptic species complex representing genospecies with different zoonotic potential or that there are at least population-specific differences in zoonotic potential [10]. However, no significant genetic differences between two *D*. *immitis* worms from Georgia (USA) and from Italy could be identified by next generation sequencing of the whole genome and comparison with microsatellite markers placed both worms within the eastern USA population [24].

In Hong Kong, *D*. *repens*-like filaria have been described as *Candidatus* D. hongkongensis and they differed considerably in their internal transcribed spacer 1 (ITS-1) sequence from the *D*. *repens* sequences deposited in GenBank [6]. Similar ITS-1 sequences have been recently identified in samples obtained from humans and dogs that acquired their infection in Europe [23, 25]. In these cases, the ITS-2 sequence from the same sample was identical to typical *D*. *repens* sequences. Therefore, cryptic species or a strong population genetic structure might be also present within *D*. *repens*.

Due to the process of global warming, summer temperatures are predicted to increase in many temperate climatic regions of the world including Central and Northern Europe. Regarding dirofilariosis, climate change will not only increase the seasonal periods of vector activity but also accelerate larval development in mosquitos and increase transmission in many geographical regions [4]. It will also lead to higher abundances of mosquitoes and prolonged activity periods [26]. Already now this allows spread of diseases currently restricted to areas with tropical or Mediterranean climate into areas with temperate climate where transmission of such diseases was unknown until recently and this trend will most likely continue in the next decades. Dogs from Central and Northern Europe accompany their owners during vacations in Mediterranean areas or they are imported from there to currently non-endemic areas e.g. by animal protection organizations aiming to provide shelter to stray dogs [27, 28]. Neither quarantines nor diagnostic tests are required for the transport of dogs between most European countries and most often not even for import from non-European countries. Moreover, not even import of animals known to carry zoonotic vector-borne pathogens is prohibited in most countries. Therefore, there is a considerable risk of endemization in regions where the infection is currently not autochthonous.

Subcutaneous/ocular dirofilariosis has been considered to be an emerging zoonotic disease in several European countries such as Russia and other former Soviet republics [29–32], Hungary [33], Poland [34] Austria [35] or France [36] but also in many tropical countries including India [37, 38]. In Germany, the first case of autochthonous human *D*. *repens* infection was reported only recently [39]. Autochthonous canine cutaneous dirofilariosis was initially reported from the Upper-Rhine area at the border to France [40, 41]. In the Berlin/Brandenburg area, *D*. *repens* positive sledge dogs were presumed to represent autochthonous cases [42, 43]. Very recently, an autochthonous canine case was postulated in central Germany [44]. However, a large survey testing more than 1000 dogs and nearly 200 foxes from Brandenburg did not identify any additional autochthonous cases of *D*. *repens* infection [25].

Due to the apparent spread of *D*. *repens*, population genetic markers are highly warranted. They could be used to identify the geographical origin of populations identified in newly endemic areas and therefore to track spreading of the disease. In addition, such markers can well be used to identify cryptic species or identify populations/genotypes that differ in their biological properties including important epidemiological determinants such as preference of certain vector species or their zoonotic potential. More detailed genetic information would also be valuable to determine if *C*. D. hongkongensis is a valid species or represents a particular *D*. *repens* population.

## Materials and Methods

### Ethics statement

Adult macrofilaria were collected during necropsy from experimentally infected dogs for a previous study and provided by Roland Schaper [45]. These animal experiments were conducted in accordance with the German Protection of Animals Act and the European Union directive 2010/63/EU and were approved by the Lower Saxonian State Office for Consumer Protection and Food Safety (LAVES) in Germany (reference number: 33.2-42502-05-LG-09-15/2012). Furthermore, DNA from adult specimen, collected by necropsy from a naturally infected dog presented at the Department of Veterinary Medicine in Bari, Italy (provided by Domenico Otranto) and from canine blood samples from Europe, which were sent in for diagnostic purposes to IDEXX laboratories (provided by Nikola Pantchev), were analyzed with the written consent of the dog owners. For the before mentioned samples sent in for diagnostic purposes to IDEXX or the Department of Veterinary Medicine in Bari, ethical approval by Institutional Animal Care and Use Committee (IACUC) or other relevant ethics board, was not required as the research was an entirely retrospective analysis of DNA samples obtained from blood and worm samples which were sent to the IDEXX Laboratories by veterinarians for routine blood examination by PCR. Otherwise, the samples were obtained and examined with the consent of the animal owners and all sample procedures were conducted in strict accordance with German and European laws. In Thailand, the study protocol was approved by the Animal Ethics Committee of Faculty of Medicine Siriraj Hospital, Mahidol University, based on the Ethics of Animal Experimentation of the National Research Council of Thailand. The Certificate of Approval number is 004/2555. All diagnostic samples were used only after anonymization of the data.

All human samples were not collected for the purpose of the present study but were removed from adult patients during surgical procedures to cure symptoms of dirofilariosis. Therefore, no approval of ethical review boards was obtained. Patient data were anonymized and all patients provided full informed written consent.

One worm was removed from the scrotum of a patient who presented at the Department of Tropical Medicine in Munich, Germany. The patient lived in Croatia for 6 month before the scrotal swelling developed. The worm sample was provided by Inge Kroidl.

Another worm was removed from the left infraocular eyelid of a patient who presented at the Department of Ophthalmology, University Medical Center Mainz, Germany. The patient had travelled in South India approximately nine months before presentation. This worm was provided by Martin Dennebaum.

In addition, DNA was obtained from a *Dirofilaria repens* removed from a human patient diagnosed in Vietnam. This case has been previously published and the DNA was provided by Thanh Hoa Le and Tran Anh Le [46].

### Generation of sequence data using PCR and Sanger sequencing

Before DNA isolation, both macrofilaria extracted from human patients were investigated microscopically. Both worms showed typical features of *D*. *repens* such as longitudinal ridges of the cuticula. The microscopical diagnosis was confirmed by determining the sequence of the 12S rRNA (*rrnS*) as previously described [15]. The sequences from the worm from the Croatian patient showed 100% homology with European *D*. *repens* isolates from dogs. The worm from the patient who travelled in India showed 100% identity to a worm isolated from another German patient, who travelled in India and was 98% identical to sequences from *D*. *repens* isolates from Europe [15].

From the human samples analyzed by next generation sequencing, DNA was isolated by first placing the sample tissue in 200 μl lysis buffer (20 mM Tris HCl pH 8.4, 100 mM KCl, 3 mM MgCl_2_, 0.9% Tween 20), then fresh DTT (2 mM final concentration) and proteinase K (20 μg) were added before incubation for 2 h at 50°C (occasionally flicking the tube with fingers). After dissolution of the tissue, 0.1 μg RNAse A (Roche) were added and the reaction was incubated at 37°C for 1 hour. Afterwards, DNA was recovered using the phenol-chloroform-method [47].

DNA was isolated from blood samples and macrofilaria using DNeasy Blood and Tissue Kit (Qiagen) or the NucleoSpin Tissue or Blood kits (Macherey Nagel) according to the manufacturer’s instructions. For blood samples from Thailand, filarial DNA was extracted from thick, dehaemoglobinized blood smears that were positive for *D*. *repens* microfilaria in a Giemsa staining as follows: Tris–EDTA buffer solution (100 μl) was added onto the blood smear slide and left for 5 min. Then the blood was scraped off the smear, transferred into a 1.5 mL microcentrifuge tube, and centrifuged for 10 min at 15,520 ×g. The Tris–EDTA buffer was discarded. The remaining sediment was kept for further DNA extraction. A high pure PCR Template Preparation Kit (Roche) was used for DNA extraction according to the manufacturer’s instructions.

Canine blood samples and adult specimens from Europe were diagnosed to be *D*. *repens* by a species-specific PCR in routine diagnostic service (IDEXX Laboratories/University Leipzig) as described previously [48]. In brief, three PCRs were conducted in parallel using the pan-filaria ITS-2 specific primers described by Rishniw et al. [49] as well as the *D*. *immitis* and the *D*. *repens* specific primers published by Favia et al. [50]. Reactions contained 1.5 mM MgCl_2_, 10 μM of each primer, 0.2 mM dNTPs and 0.75 U GoTaq Flexi DNA Polymerase (Promega) in 25 μl 1 × Colorless GoTaq Flexi Buffer. After initial denaturation at 95°C for 2 min, PCR products were separated on 1.5% agarose gels and the banding patterns were used to identify *D*. *immitis*, *D*. *repens* and other canine filarial species as single or mixed infections. Canine samples from Thailand were identified to be *D*. *repens* positive using a high-resolution melt PCR targeting the cytochrome oxidase subunit 1 primers described by Rishniw et al [49].

For amplification of overlapping mitochondrial genome sequences, PCR primers were chosen from regions with high identity between mitochondrial genome sequences of *D*. *immitis*, *Onchocerca volvulus* and *Onchocerca flexuosa*. Moreover, it was aimed to produce overlapping PCR products with a size of approximately 2500 bp. All PCRs were conducted using Phusion Hot Start II High Fidelity DNA polymerase (Thermo Scientific). Reactions contained 0.02 U/μl polymerase, 0.2 μM dNTPs, 0.5 μM of each primer, 1 M betaine (SigmaAldrich) in 50 μl 1× Phusion HF buffer. Reactions were denatured at 98°C for 2 min, followed by 50 cycles of 98°C for 5 s, a primer-pair specific annealing temperature for 30 s and 72°C for 2 min. Primer pairs and annealing temperatures are shown in S1 Table. Amplification products were analyzed by agarose gel electrophoresis, purified from gels not exposed to UV light using the Gel DNA recovery kit (Zymo Research) and cloned into the pSC-B-amp/kan vector (StrataClone Blunt Cloning Kit, Agilent Technologies). Clones with inserts were sequenced by primer walking at LGC Genomics (Berlin). All clones were completely sequenced on both strands. Final assembly of the mitochondrial genome from individual reads was conducted in CloneManager 9 software (Scientific & Educational Software).

### Sequencing of mitochondrial genomes using MiSeq technology

Mitochondrial genome sequences were essentially obtained as described previously by Besnard et al. [47]. In brief, libraries were constructed using Illumina Nextera XT kits according to manufacturer’s instructions and sequenced on an Illumina MiSeq using v3 chemistry (Illumina, Netherlands) to obtain 300 bp paired end reads. Assembly was performed using the CLC Genomics Workbench 7.0.4 with parameters that were slightly different for the three samples and as detailed in S1 Supporting Information, S2 Supporting Information and S2 Table.

### Sequence annotation and analyses

The mitochondrial genomes were annotated by identifying open-reading frames followed by local alignment with proteins encoded in the mitochondrial genomes of other spirurida with particular focus on *D*. *immitis*. Then, tRNAs were predicted using the tRNAscan-SE 1.21 [51, 52] with the parameters optimized for nematode mitochondrial tRNAs and Arwen [53] software. Ribosomal RNA genes were identified by comparison with the *D*. *immitis*, *O*. *flexuosa* and *O*. *volvulus* mitochondrial genome using BLASTn searches. Codon usage data for *D*. *repens* and *D*. *immitis* were obtained by analysis using the sequence manipulation suite [54].

AT and GC skews of the coding strand were calculated as [(frequency (A)–frequency (T)]/[frequency (A) + frequency (T)] and [(frequency (G)–frequency (C)]/[frequency (G) + frequency (C)], respectively.

### Phylogenetic analyses

For phylogenetic analyses, protein and rRNA coding sequences of the mitochondrial genomes of other spirurids were retrieved from GenBank: *D*. *immitis* (AJ537512), *O*. *volvulus* (AF015193), *O*. *flexuosa* (HQ214004), *Wuchereria bancrofti* (JN367461, JF775522, JQ316200, HQ184469), *Brugia malayi* (AF538716), *Chandlerella quiscali* (HM773029), *Acanthocheilonema vitae* (HQ186249), *Setaria digitata* (GU138699), *Heliconema longissimum* (GQ332423), *Spirocerca lupi* (KC305876), *Thelazia callipedia* (JX069968) and *Gongylonema pulchrum* (KM264298) were included. The ascarid *Ascaris lumbricoides* (JN801161) and the medina worm *Dracunculus medinensis* (JN555591) were chosen as outgroups.

Protein-coding DNA sequences were initially aligned codon-wise according to the conceptual translation (invertebrate mitochondrial genetic code) with MUSCLE [55] in Mega 6 [56]. Using DAMBE 5.5.29 software [57] a test for substitution saturation [58, 59] was performed separately for first and second codon positions on one hand and third codon positions on the other hand. Alignments of protein sequences was conducted using the M-COFFEE mode of T-COFFEE 11.00 [60, 61] and rRNA sequences were aligned using MAFFT with the Q-INS-i option to include predicted secondary structure information in the alignment optimization [62, 63]. For each protein, optimal substitution models were identified using ProtTest 3 [64, 65]. The number of substitution rate categories was set to 25. A supermatrix of alignments was generated in the FASconCAT-G [66] software. Finally, a maximum-likelihood analysis with data partitioned according to the different genes was performed using RAxML 8.1.11 [67] on the CIPRES Science Gateway server [68]. The MtArt +I+G+F model was used for the partitions representing *nduo-1*, *nduo-4*, the MtArt +G+F model for *ctb-1*, *ctc-1*, *ctc-3*, the JTT +G+F model for *atp-6*, *ctc-2*, *nduo-2*, *nduo-3*, *nduo-5*, *nduo-6* and the LG+I+G+F model for *ndfl-4* as well as the DNA +G model for both rRNA genes. Initially, the rapid bootstrapping algorithm was used to identify the tree with the highest likelihood and to obtain branch support values in a single run. In a second step, a Shimodaira-Hasegawa (SH) likelihood ratio test (LRT) was conducted in RAxML with the optimal tree obtained in the bootstrapping run as additional input to calculate an independent estimate for branch support in this tree. The resulting phylogram was visualized in Mega 6 and rooted on the outgroup.

For analysis of phylogenetic relationship between *D*. *repens* like samples, the sequences were aligned using MAFFT in the G-INS-I mode [69] before jmodeltest 2.0 was used to identify a suitable substitution model [70]. Since no clearly preferable model could be identified, the parameter-rich GTR +I+G+F model was chosen. The phylogenetic tree was calculated using PhyML 3.1 [71] with the number of Γ rate categories set to 25 and the α shape parameter of the Γ distribution was optimized by PhyML. For tree optimization, the best of nearest-neighbor interchanges and subtree-prune-regraft moves was allowed. Two distinct runs, each starting with a neighbor-joining and five random trees, were conducted and both runs converged on the same tree topology. Branch support was calculated using the SH as well as a Bayesian transformation of the LRT.

### Identification of variable regions in filarial mitochondrial genomes

To identify regions with high variability within the Onchocercidae or within *D*. *repens*-like sequences, a sliding window analysis was performed. In order to avoid that species for which more than one mitochondrial genome sequence is available exert a stronger influence on the analysis than other species, only one mitochondrial genome of *D*. *repens* (Accession no KX265047) and *W*. *bancrofti* (JN367461) was included in the alignment together with genomes from *D*. *immitis*, *O*. *volvulus*, *O*. *flexuosa*, *B*. *malayi*, *C*. *quiscali*, *L*. *loa* and *A*. *vitae* using MUSCLE [55] as implemented in MEGA 6.06 [56]. In order to estimate local nucleotide diversity (π) using DnaSP 5.10.01 [72], a sliding window of 300 bp was moved in steps of 10 bp over the entire alignment and π was calculated as Jukes & Cantor distance. Indels were excluded using DnaSP. Nucleotide diversity was plotted against mid-point positions of the sliding window in GraphPad Prism 5. An identical analysis was conducted comparing only the four mitochondrial genomes from *D*. *repens* like worms identified in the present study.

## Results and Discussion

### Annotation of the *D*. *repens* mitochondrial genome

In the following, all genes are designated strictly according to the nomenclature for *Caenorhabditis elegans* as suggested by Beech et al. [73]. In order to facilitate comparisons, Table 1 also list names that are more frequently used in the parasitological literature.

**Table 1:** Annotated genes in the mitochondrial genome of *Dirofilaria repens*.

Gene	Alt. name^a^	Position	Size (bp)^b^	Number of amino acids	Init./term./anti-codon^c^	Type of tRNA	Intergenic nucleotides^d^
*trnE*		3–58	56		TTC	TV loop	-1
*trnS*^AGN^		58–108	51		TCT	D loop	-4
*nduo-2*	nadh-2	105–959	855	284	ATT/TAA		9
*trnT*		969–1025	57		TGT	TV loop	-1
*nduo-4*	nadh-4	1025–2254	1230	409	TTG/TAA		7
*ctc-1*	cytc	2262–3908	1647	548	ATT/TAA		9
*trnW*		3918–3975	58		TCA	TV loop	47
*nduo-6*	nadh-6	4023–4475	453	150	TAT/TAA		2
*trnR*		4478–4532	55		ACG	TV loop	7
*trnQ*		4540–4593	54		TTG	TV loop	6
*ctb-1*	cytb	4600–5680	1081	360	ATT/T		0
*trnL*^CUN^		5681–5735	55		TAG	TV loop	0
*ctc-3*	coIII	5736–6515	780	259	ATT/TAA		
CR^e^	AT-rich^e^	6516–6796	284				
*trnA*		6797–6853	57		TGC	TV loop	0
*trnL*^UUR^		6854–6907	54		TAA	TV loop	-1
*trnN*		6907–6966	60		GTT	TV loop	3
*trnM*		6970–7027	58		CAT	TV loop	0
*trnK*		7028–7084	57		CTT	TV loop	1
*ndfl-4*	nadh-4L	7086–7328	243	80	GTA/TAA		0
*rrnS*		7329–8008	680				0
*trnY*		8009–8062	54		GTA	TV loop	-3
*nduo-1*	nadh-1	8060–8958	898	299	TTG/T		-22
*trnF*		8937–8995	59		GAA	TV loop	3
*atp-6*		8999–9574	576	191	ATT/TAG		9
*trnI*		9584–9640	56		GAT	TV loop	20
*trnG*		9661–9718	58		TCC	TV loop	3
*ctc-2*	coII	9722–10418	697	232	ATT/T		0
*trnH*		10419–10473	55		GTG	TV loop	0
*rrnL*		10474–11432	959				0
*nduo-3*	nadh-3	11434–11770	337	112	CTT/T		1
*trnC*		11771–11826	56		GCA	TV loop	0
*trnS*^UCN^		11827–11882	56		TGA	D loop	0
*trnP*		11886–11946	60		TGG	Clover leaf	3
*trnD*		11949–12008	60		GTC	TV loop	2
*trnV*		12009–12065	57		TAC	TV loop	0
*nduo-5*	nadh-5	12069–13661	1593	530	TTG/TAG		13

^a^Alternative name not corresponding to the nomenclature in *Caenorhabitis elegans*.

^b^Size including (partial) stop codons.

^c^Initiation/termination of protein coding genes; anti-codon of tRNA genes

^d^Number of nucleotides between this gene and the following gene. Negative numbers indicate overlapping genes.

^e^AT-rich control region

Complete mitochondrial DNA sequences were obtained from three European and one Indian sample diagnosed initially as *D*. *repens*. For one of the European worms, six overlapping PCR fragments were cloned, sequenced and assembled. Raw data of MiSeq reads obtained for each of the three other worms are summarized in S3 Table. Assembled sequences were deposited in GenBank under the accession numbers KX265047- KX265050. The total length of the sequences varied between 13,672–13,680 bp which is slightly smaller than the 13,814 bp mitochondrial genome of *D*. *immitis*.

All four sequences were compared to database entries for *ctc-1*, *rrnS* and *rrnL* to confirm the worm species. The three European worms showed 99–100% identity to *D*. *repens* entries in the *ctc-1*, 95–100% at the *rrnS* and 97–98% at the *rrnL* locus with most of the comparisons showing >98% identity. These samples were therefore considered to represent *D*. *repens* in the following. The Indian sequence showed 100% identity to the only *ctc-1* entry for *C*. D. hongkongensis, and it was 99% identical to an unpublished *C*. D. hongkongensis *rrnS* sequence kindly provided by Kwok-Yung Yuen (University of Hong Kong). The latter sequence was obtained from the same material used for the original description of *C*. D. hongkongensis [6]. It was also 99% identical to an *rrnS* gene sequence published previously for a *D*. *repens* from India [15]. The perfect identity to the *ctc-1* and the very high identity to the *rrnS* sequences from the original report of *C*. D. hongkongensis identified this sample as a representative of this proposed species. Otherwise, the Indian sequence similarity with *ctc-1*, *rrnS* and *rrnL* of *D*. *repens* entries was 95–96%, 95–98% and 99%, respectively.

Pairwise identities of whole genome sequences as determined by Blastn were 99% between the four European *D*. *repens* sequences (S4 Table, green labeled fields), including a *D*. *repens* mitochondrial genome sequence (KR071802) recently deposited in Genbank (09-April-2016), which is not yet linked to a publication. S4 Table clearly shows that all comparisons between known species show identities between 84 and 89% (labeled red) with two exceptions: First, identity between any *D*. *repens* and the Indian *C*. D. hongkongensis sequence was 94%. Secondly, the comparison between *O*. *volvulus* and *Onchocerca ochengi* (yellow) (unpublished mitochondrial genome sequence available at http://www.nematodes.org/genomes/onchocerca_ochengi/ with kind permission of Mark Blaxter and Georgios Koutsovoulos, University of Edinburgh) was 97%. Since the two sibling *Onchocerca* species show fewer differences than *D*. *repens* and *C*. D. hongkongensis, it can be assumed that the latter are also two valid species.

**Fig 1:**
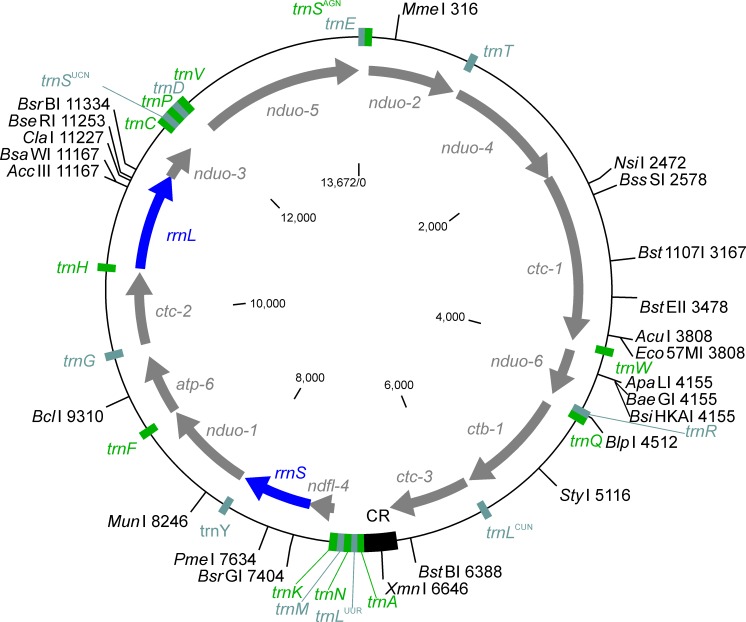
Schematic map of the 13,672 bp *Dirofilaria repens* mitochondrial genome. On the outer circle, positions of restriction enzymes recognition sites (with positions in the genome) are indicated for enzymes that cut only once in the genome. The position of tRNA genes and the AT-rich control region (CR) are also shown on this circle. The next circle shows the position of the genes encoding proteins (gray) or rRNAs (blue). In the center, the positions on the map is provided in base pairs (bp). All genes, including the genes encoding rRNAs are orientated in clockwise direction. The map was drawn using the sequence with the accession no KX265047.

The following detailed description refers to the *D*. *repens* sequence (accession no: KX265047) and the *C*. D. hongkongensis sequence (KX265050). Annotation identified genes encoding twelve proteins, two rRNAs and 22 tRNA genes (Table 1) which are all located on the same strand and arranged in the same manner as in the mitochondrial genomes of other parasites from the family Onchocercidae (Fig 1). An *atp-8* gene is missing as in all spirurida and other more derived nematode groups. In contrast, the *atp-8* gene is present in mitochondrial genomes of clade I nematodes such as the trichocephalids *Trichinella* and *Trichuris* [74, 75].

The protein and rRNA coding genes show a varying degree of conservation between *D*. *repens* and *C*. D. hongkongensis as well as with *D*. *immitis* (S5 Table). For all genes, identity and similarity are much higher for comparisons of *D*. *repens* with *C*. D. hongkongensis than for comparisons between these parasites and *D*. *immitis*. Identities between *D*. *repens* and *C*. D. hongkongensis range between 90.7 and 97.5% on the DNA and 85.5 and 100% on the amino acid level. Comparisons including *D*. *immitis* range between 83.2 and 90% on DNA and 76.1 and 95.1% on the amino acid level. In particular, *atp-6* and some of the NADH reductase subunits such as *nduo-3*, *nduo-2* and *nduo-6* show only a moderate degree of conservation.

In S1 Fig and S2 Fig, codon usage and amino acid frequencies are compared between *D*. *immitis* and *D*. *repens*, the members of the genus with the lowest and highest T content on the coding strand. The absolute number of codons and relative synonymous codon usage (RSCU) as well as the frequency of amino acids are shown. In general, both patterns are very similar but the extremely low AT skew is associated with an even stronger preference of T-rich codons in *D*. *repens*. In comparison to codon usage, amino acid frequencies show only very small differences (S2 Fig). For detailed description of nucleotide composition content and nucleotide skews in the coding strand, non-coding regions and use of different stop and start codons see S3 Supporting Information.

### Phylogenetic analysis

**Fig 2:**
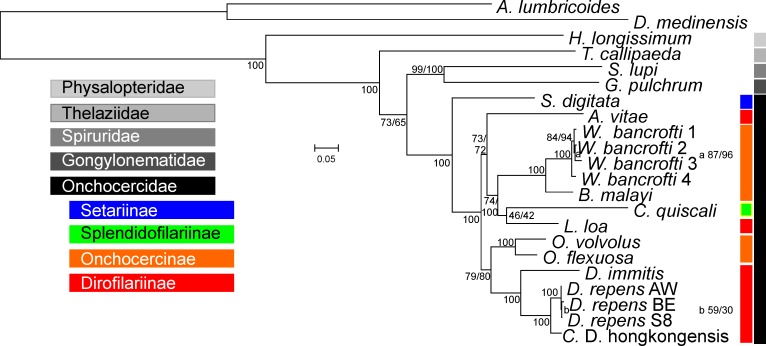
Phylogenetic analysis of spirurid nematodes using complete mitochondrial genomes. The mitochondrial genomes of *Dirofilaria repens* (from top to bottom: KX265048, KX265047, KX265049), *Dirofilaria hongkongensis* (KX265047), *Dirofilaria immitis*, *Onchocerca volvulus*, *Onchocerca flexuosa*, *Wuchereria bancrofti* (from top to bottom: JN367461, JF775522, JQ316200, HQ184469), *Brugia malayi*, *Chandlerella quiscali*, *Acanthocheilonema vitae*, *Setaria digitate*, *Heliconema longissimum*, *Spirocerca lupi*, *Thelazia callipedia* and *Gongylonema pulchrum* were included in the analysis. The mitochondrial genomes of *Ascaris lumbricoides* and *Dracunculus medinensis*, clade III parasitic nematode not belonging to the Spirurida were used as outgroup. Concatenated alignments were analyzed with RAxML 8.1.11 with optimized models for each gene partition using the rapid bootstrapping algorithm. The Shimodaira-Hasegawa likelihood ratio test as implemented in RAxML was used as an alternative, unrelated method to obtain support values for all individual internal notes of this tree. Support values obtained by bootstrapping and by the likelihood ratio test are provided before and after the slash. If only one number is shown, the results of the tests were identical. Small letters were used if the space was too limited to write the support values directly at the branch. At the right of the scheme, different “classical” families within the Spirurida as defined by morphological criteria [81] are indicated as bars in different gray scales. As colored bars, different “classical” subfamilies within the Onchocercidae are indicated.

A phylogenetic tree was constructed by multi-locus phylogenetic analysis. Initially, all protein coding nucleic acid sequences were mapped in order to construct codon-wise alignments of the coding sequences. Then, a test for substitution saturation [58, 59] was conducted for all protein coding genes separately for (i) the first and second and (ii) the third codon position. This assay revealed significant saturation at the third codon position. Therefore, protein-coding genes were analyzed on the amino acid and not the nucleic acid level. After alignment of amino acid sequences, optimal substitution models were identified in ProtTest 3. Protein and rRNA gene alignments were concatenated and a maximum likelihood tree was calculated with optimized substitution models for each gene partition in RAxML using the rapid bootstrapping option. Node-support was obtained from bootstrapping values and SH LRTs. Fig 2 shows the tree obtained from the currently available complete mitochondrial genomes of spirurid nematodes. The tree topology is well in agreement with recently published results using similar species combinations [76–78] and multi-locus phylogenetic analysis including both mitochondrial and nuclear loci [79]. However, the tree topology is in disagreement with the classical systematic of Onchocercidae as described by Anderson and Bain [80] and Bain et al. [81]. The Thelaziidae are apparently located basal in the Spirurida. This group has very high support of 95% and 99% by bootstrapping and the SH LRT test, respectively. The next branch carries the species *G*. *pulchrum* and *S*. *lupi* (support by bootstrapping and the SH test was 99% and 100%, respectively). Gongylonematidae and Spiruridae are both placed in the superfamily Spiruroidea [81].

Within the Onchocercidae, which are shown to be monophyletic in the analysis as they are strongly supported with both tests (100%), *S*. *digitata* is placed at the most basal position of all species included in the present analysis. It is the sister operational taxonomic unit to a highly supported group (100% in both tests) containing the only included member of the Splendidofilarinae (*C*. *quiscali*) and all Onchocercinae as well as Dirofilarinae. Within this group, tree topology does not at all represent the allocation of the species to the different subfamilies. Operational taxonomic units with very high statistical support (100%) are the genera *Onchocerca* and *Dirofilaria* and a group comprised of the lymphatic filaria *W*. *bancrofti* and *B*. *malayi*. *L*. *loa* is generally considered to belong to the subfamily Dirofilarinae but is not placed as sister group to *Dirofilaria* in the current analysis. The members of the Onchocercinae are even more scattered over the tree. The genus *Onchocerca* appears to be most closely related to *Dirofilaria* with a good support in bootstrapping (79%) and the SH test (80%). The lymphatic filaria are within a very heterogeneous group which also contains *C*. *quiscali* (Splendofilarinae) and *L*. *loa* (Dirofilarinae). *A*. *vitae* (Onchocercinae) is located at the basal position within this group.

The three *D*. *repens* sequences form a group with high statistical support of 100% in both types of analyses. With the same statistical support, *D*. *repens* and *C*. D. hongkongensis are placed together as closely related operational taxonomic units. The difference between the *D*. *repens* and *C*. D. hongkongensis sequences is much larger than between the four *W*. *bancrofti* sequences in the analysis but still smaller than the differences between *D*. *repens* and *D*. *immitis*, *C*. D. hongkongensis and *D*. *immitis* or between *O*. *volvulus* and *O*. *flexuosa*, the only other pairs in the analysis coming from the same genus. This suggests that *D*. *repens* and *C*. D. hongkongensis are very closely related. Due to the large sequence length used here, these results confirm that *C*. D. hongkongensis is more closely related to *D*. *repens* than to *D*. *immitis* as already suggested by *ctc-1* analysis presented by To et al. (6). However, their analysis using 18S with ITS-1 or only ITS-1 sequences were in contradiction to this view. The fact that ITS-1 sequences very similar to *C*. D. hongkongensis ITS-1 sequences can be found in European *D*. *repens* probably explains this discrepancy. Nevertheless, the final description of phylogenetic relationships between members of the genus *Dirofilaria* will require inclusion of additional genus members and additional loci such as larger fragments of single-copy nuclear genes.

### Mitochondrial genome variability

**Fig 3:**
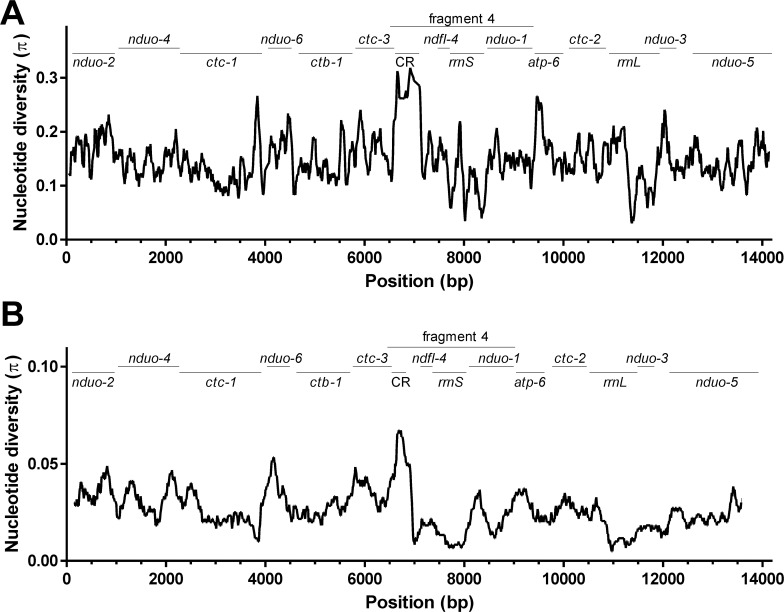
Analysis of sequence variability along the sequence of the mitochondrial genomes of Onchocercidae. (A) All sequences of mitochondrial genomes from Onchocercidae were aligned with MUSCLE and nucleotide diversity was determined in DnaSP using a Jukes-Cantor corrected distance. In order to avoid that the result is skewed due to overrepresentation of a single species, only one of four *Wuchereria bancrofti* (JN367461) and *Dirofilaria repens* genomes (KX265047) were used. Nucleotide diversity was calculated for a 300 bp window and plotted against the mid-point of this window. The window was moved in 10 bp steps across the alignment. Since the software is not able to handle circular DNA molecules, results at the beginning and the end of the alignment should be considered with care. Above the plot, the position of the different protein and rRNA coding genes as well as the AT-rich control-region (CR) are indicated. The PCR fragment 4, which was selected for further analysis in a larger set of samples from different geographical origins is shown at the top of the figure. (B) The same analysis was carried out comparing the three *D*. *repens* and the *C*. D. hongkongensis mitochondrial genomes (KX265047- KX265050).

In mitochondrial genomes, regions with high variability between closely related species have also been proposed to be those regions where presumably most intraspecific variability occurs [82]. In order to identify such regions in mitochondrial genomes of Onchocercidae, an alignment with sequences of all nine members of the Onchocercidae for which complete mitochondrial genome sequences are available was used to determine nucleotide diversity with a 300 bp sliding window analysis. Nucleotide diversity π was plotted against the midpoint of the window (Fig 3A). Nucleotide diversity varied widely between 0.03 and 0.34 (median 0.14). Not unexpectedly, the highest diversity is found in the largest non-coding region of the genomes, the AT-rich CR. Additional peaks of diversity were identified in the genes encoding *atp-6*, *ctc-1* and *nduo-3*. Regions with low diversity were found in *ctc-1*, *rrnS* and *rrnL*.

Using the three complete *D*. *repens* and the *C*. D. hongkongensis mitochondrial genome sequences, the same approach was used to identify regions with high variability in the *D*. *repens* like parasites (Fig 3B). The observed diversity within the 300 bp windows (median 0.026, range 0.005–0.067) was much smaller than for the analysis over all Onchocercidae (p<0.0001 in a Mann-Whitney U test). Remarkably, however, the fold difference between the region showing the highest diversity to the region with the lowest diversity is relatively similar with 10.5 fold in the analysis using all Onchocercidae and 13.4 fold in the comparison of *D*. *repens* like parasites. Again, the non-coding CR shows a strong peak of diversity followed by a region in the *nduo-6* gene.

### Survey of intraspecific variability in European and Asian *D*. *repens* like samples

Aiming to identify genetic differences between *D*. *repens* populations, fragment 4 was chosen for comparative sequence analysis from a larger number of samples. This fragment on one hand contains the CR showing the highest intraspecific diversity but also the *rrnS* and *nduo-1* genes with only moderate variability. For *rrnS*, there are also many entries available in GenBank for nematode species and the ribosomal genes do not suffer from as rapid substitution saturation as third codon positions in protein coding genes.

The fragment was amplified from 41 canine blood samples collected from different European countries (n = 39) and Thailand (n = 2). Furthermore, a sequence from a macrofilaria collected from a human patient in Vietnam as well as the corresponding sequences from the four complete mitochondrial genomes were included. Within these 47 sequences, 41 different haplotypes were identified. The alignment consisted of 2588 sites with 214 polymorphic sites and 65 gaps showing variation between the sequences. On average, there were 21.7 nucleotide differences between two sequences.

**Fig 4:**
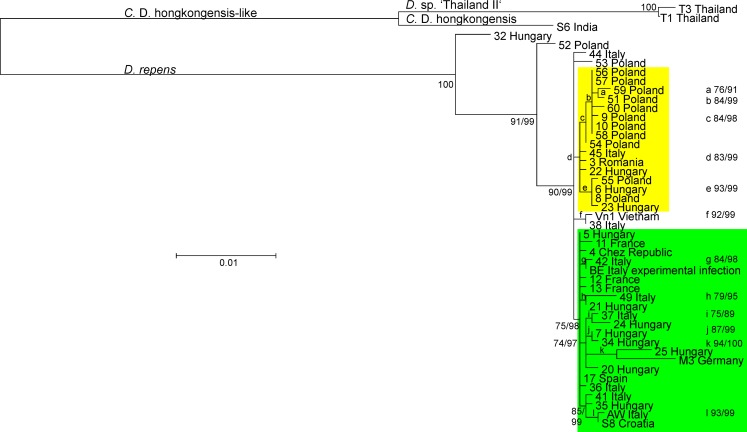
Phylogenetic analysis of *Dirofilaria* repens like sequences from samples collected in Europe and Asia. Sequences of an approximately 2.5 kb mitochondrial DNA fragment including parts of the *ctc-3* and the *trnF* genes, the AT-rich control region, the complete protein coding genes for *ndfl4* and *nduo-1*, the ribosomal RNA gene *rrnS* and the genes for the tRNAs *trnA*, *trnL*^UUR^, *trnN*, *trnM* and *trnK*. Sample origin in terms of countries are shown behind an in-house sample number/code. All sequences have been deposited in GenBank under the accession nos. KX265047—KX265093. In addition, samples of human origin are indicated, all other samples were from dogs. Numbers at the internal nodes represent results for the Shimodaira-Hasegawa and a Bayesian transformation of the likelihood ratio test before and after the slash, respectively. If only one number is shown, the results of the tests were identical. Small letters were used if the space was too limited to write the support values directly at the branch. The branches labelled with “*Dirofilaria repens*”, “*Candidatus* Dirofilaria (*C*. D.) hongkongensis” and *Dirofilaria* (*D*.) sp. ‘Thailand II’ contain only the respective (putative) species. The branch labeled with “*Candidatus* Dirofilaria (*C*. D.) hongkongensis like” clusters together this proposed species with sequences of *C*. D. sp. ‘Thailand II’ that might belong to the same species or represent an additional species. The yellow area indicates all sequences that belong to an apparently monophyletic group containing a large number of samples from Poland and Hungary. The green color labels a second apparently monophyletic group with most samples coming from South-Western Europe and Hungary.

A phylogenetic tree was constructed using PhyML 3.1. In this unrooted tree, three clusters were defined by separating groups connected by long branches (Fig 4). The largest cluster contains all *D*. *repens* sequences including all samples from Europe and the one from Vietnam. The second “cluster” consists only of the *C*. D. hongkongensis sequence from India. The third cluster contains both closely related sequences from dogs in Thailand. This might represent a third species (*Candidatus* Dirofilaria sp. ‘Thailand II’) or another *C*. D. hongkongensis genotype. Therefore, the branch leading to this group was labeled “*C*. D. hongkongensis like”. It must be emphasized that the *C*. D. sp. ‘Thailand II’ is presumably not identical to *Dirofilaria* sp. ‘Thailand’ (NCBI taxonomy ID: 1571575). For the latter, only a single GenBank entry (KM374817) for an ITS-1 sequence is available. A partial ITS-1 sequence for sample T1 revealed only 83% identity with KM374817.

Within the *D*. *repens* cluster, two samples (sample IDs 32 from Hungary and 52 from Poland) showed a larger difference to the remaining *D*. *repens* sequences and were located basally in the *D*. *repens* group (Fig 4). Except for these two sequences, the differences between the *D*. *repens* samples were in general rather low. There were three groups with moderate to good support, depending on the test, and two sequences that could not be allocated to any of the groups (44 Italy and 53 Poland). One very small group contained only the sequence from Vietnam (Vn1) and one Italian sequence (38 Italy). The first of the larger clusters, highlighted by the yellow area in Fig 4, contains predominantly sequences obtained from Polish samples (11 out of 16 in the cluster). The remaining 5 samples in this cluster were from Hungary (3), Italy and Romania. The second large cluster with considerable statistical support is highlighted by the green area in Fig 4. This cluster predominantly contains samples from Western Europe such as Italy (7), France (3), Spain, Croatia and the Chez Republic but also from Hungary (8). The sample from the outbreak in Brandenburg (M3 Germany) also falls into this cluster. Samples from Hungary show the highest genetic diversity and can be found all over the *D*. *repens* tree. The two samples from human patients do not form a separate cluster providing no hints that only certain *D*. *repens* genotypes might be involved in human infections although the number of human samples included is far too small to allow definitive conclusions. It should also be mentioned that the origin of the dog samples from certain countries does not guarantee that these dogs have been infected there. At least some of them might have been infected during traveling abroad.

Although only four samples from Asia were included in the present study, Fig 4 clearly shows that the differences within the European samples are much smaller than in the Asian samples. One of the Asian samples (Vn1 Vietnam) was clearly a *D*. *repens* genotype and grouped into the cluster of European samples. This confirms that *D*. *repens* definitively has a wide geographic distribution including South-East Asia. In contrast, there was no evidence of *C*. D. hongkongensis genotypes in the European *D*. *repens* population despite the large number of samples analyzed from Europe. Even within the *C*. D. hongkongensis like group (Fig 4), variability is much higher between the Indian and the Thai sequences than variability within the European *D*. *repens* sequences. This suggests that there might be several unrecognized genotypes or even cryptic species that are usually identified as *D*. *repens* in routine diagnosis.

### How many valid species belong to the *D*. *repens* species complex?

To determine, if *C*. D. hongkongensis or any of the other genotypes in the *D*. *repens* complex such as *Dirofilaria* sp. ‘Thailand II’, *Dirofilaria* sp. MK-2010 (NCBI Taxonomy ID: 866281) and *Dirofilaria* sp. ‘Thailand’ (NCBI Taxonomy ID: 1571575) in GenBank belong to different valid species or are only representatives of different populations within *D*. *repens* (or *C*. D. hongkongensis), a much larger number of samples from these populations would be required. Since mitochondrial genomes are generally considered to be inherited only maternally in nematoda, their genetic information is not suitable to detect genetic recombination or events such as hybridization between species, subspecies or genotypes. In the genus *Onchocerca*, there are four closely related mitochondrial genotypes, *O*. *volvulus*, *O*. *ochengi*, *Onchocerca dukei* and *Onchocerca* sp. “siisa”. *O*. *volvulus* and *O*. *ochengi* are clearly valid species using different definitive hosts although the overall nucleotide identity between their mitochondrial genomes is 97% compared to 94% for *D*. *repens* and *C*. D. hongkongensis. Although the mitochondrial genotype of *Onchocerca* sp. “siisa” appears to be at least as closely related to *O*. *volvulus* as to *O*. *ochengi*, if not closer to *O*. *volvulus*, there is free gene flow between *O*. *ochengi* and *Onchocerca* sp. “siisa” as revealed by analysis of nuclear genes. Females and males of both genotypes apparently mate without any restrictions producing viable microfilariae [83, 84]. Therefore, *Onchocerca* sp. “siisa” must be considered a particular mitochondrial genotype of *O*. *ochengi* and does not represent a distinct species. In addition to mitochondrial genetic information, sequences from nuclear genes are required to detect if genetic exchange between such genotypes is possible and occurs within natural populations. Preliminary comparisons of *D*. *repens*, *C*. D. hongkongensis, *Dirofilaria* sp. ‘Thailand II’, *Dirofilaria* sp. ‘Thailand’ and *Dirofilaria* sp. MK-2010 suggest that ITS sequences are not very suitable for this purpose. Multiple single copy genes would provide a more precise information relying exclusively on one-to-one orthologues which is not possible for multi-copy ribosomal genes. The low genetic diversity in Europe compared to the Asian samples suggests that Asia is a hot spot for genetic diversity within *D*. *repens* like parasites and that multiple genotypes might co-exist there while only one genotype was introduced into Europe.

### Conclusions

The study provides important genetic information about diversity of zoonotic *Dirofilaria* species causing cutaneous forms of disease in dogs. Diversity of these agents is apparently larger than anticipated over decades and additional species including *C*. D. hongkongensis but maybe also *Dirofilaria* sp. ‘Thailand II’ obviously contribute to the infection risk at least in tropical regions of Asia. Since emerging resistance to metaphylactic treatment with macrocyclic lactones is an important issue regarding *D*. *immitis* in the U.S.A., a similar problem might also arise for *D*. *repens* and related pathogens in areas with high treatment frequencies to either prevent heartworm infections or reduce the burden of zoonotic infections in humans. If this occurs, it would be important to know the number of valid, genetically isolated species in this complex and have more population genetic information to predict the risk of spread of resistant phenotypes between regions.

## Supporting Information

S1 Table PCR primer sequences and annealing temperatures. (PDF)

S2 Table Mapping and assembly parameters for CLC Genomics Workbench 7.0.4. (PDF)

S3 Table MiSeq read information. (PDF)

S4 Table Nucleotide identity (%) in pairwise comparisons between complete mitochondrial genomes of members of the genera *Dirofilaria* and *Onchocerca*. (PDF)

S5 Table Nucleotide identity (%) and amino acid identity (similarity) (%) in pairwise comparisons between protein and rRNA coding genes in the mitochondrial genomes of *Dirofilaria repens*, *Candidatus* Dirofilaria hongkongensis (*C*. D. hong.) and *Dirofilaria immitis*. (PDF)

S1 Fig Comparison of codon usage between *Dirofilaria repens* and *Dirofilaria immitis*. For every amino acid as well as for Start and Stop codons the absolute number (A) and the relative synonymous codon usage (i.e. number of codons divided by the frequency expected if all synonymous codons would occur with equal frequencies) (B) are shown as stacked bar plots. For every amino acid, the left bar shows values for *D*. *repens* and the right bar for *D*. *immitis*. The codons encoding the different amino acids are numbered and color-coded as indicated in (C).
(PDF)

S2 Fig Amino acid composition of the proteins encoded in the mitochondrial genomes of *Dirofilaria repens* and *Dirofilaria immitis*. Absolute numbers of amino acids are given with frequencies in the genome in brackets.
(PDF)

S1 Supporting Information Assembly details for genomes from Illumina reads. (PDF)

S2 Supporting Information Shell script to extracted paired partner reads to reads mapped to the *D*. *immitis* mitochondrial genome. (SH)

S3 Supporting Information Description of nucleotide composition content and nucleotide skews in the coding strand, non-coding regions and use of different stop and start codons. (PDF)
