# Correction: The Mitochondrial Genomes of the Zoonotic Canine Filarial Parasites *Dirofilaria* (*Nochtiella*) *repens* and *Candidatus* Dirofilaria (Nochtiella) Honkongensis Provide Evidence for Presence of Cryptic Species

**DOI:** 10.1371/journal.pntd.0008347

**Published:** 2020-05-26

**Authors:** Esra Yilmaz, Moritz Fritzenwanker, Nikola Pantchev, Mathias Lendner, Sirichit Wongkamchai, Domenico Otranto, Inge Kroidl, Martin Dennebaum, Thanh Hoa Le, Tran Anh Le, Sabrina Ramünke, Roland Schaper, Georg von Samson-Himmelstjerna, Sven Poppert, Jürgen Krücken

The title currently is The Mitochondrial Genomes of the Zoonotic Canine Filarial Parasites *Dirofilaria* (*Nochtiella*) *repens* and *Candidatus* Dirofilaria (Nochtiella) Honkongensis Provide Evidence for Presence of Cryptic Species. The title contains spelling errors and should be corrected to: The Mitochondrial Genomes of the Zoonotic Canine Filarial Parasites *Dirofilaria* (*Nochtiella*) *repens* and *Candidatus* Dirofilaria (Nochtiella) hongkongensis Provide Evidence for Presence of Cryptic Species.

The affiliation of the 10^th^ author, Tran Anh Le, is currently Department of Parasitology, Viet Nam Veterinary Medical University, Ha Noi, Viet Nam. The correct affiliation of the 10th author, Tran Anh Le, is Department of Parasitology, Vietnam Military Medical University, Hanoi, Vietnam.

In the manuscript, there is reference to canine blood samples used to amplify partial mitochondrial DNA sequences of the putative species Dirofilaria sp. "Thailand II". This is incorrect. It should be noted that the samples came from cats and not dogs.
