# Supplementary material for: The Mitochondrial Genomes of the Zoonotic Canine Filarial Parasites Dirofilaria (Nochtiella) repens and Candidatus Dirofilaria (Nochtiella) Honkongensis Provide Evidence for Presence of Cryptic Species
Source: PLoS Negl Trop Dis. 2016 Oct 11;10(10):e0005028. doi: 10.1371/journal.pntd.0005028 (PMC5058507; doi:10.1371/journal.pntd.0005028)
Supplement: S1 Table — (PDF) [file pntd.0005028.s001.pdf]

S1 Table. PCR primer sequences and annealing temperatures

Primer name	Primer sequence (5'→3')	Annealing temperature
Drep1for	TATTTTTAATTTTCGATTAAAAGATT	57 °C
Drep1rev	AAAACAGAATTATAAACCTGACCA	
Drep2for	GGTTTATTTTTGTTATTTAGTATGAA	54 °C
Drep2rev	TATACTTCCAAAATTCCATATATAACT	
Drep3for	TTACTATATCTGATTACGGCTCAGCTAGAATT	64 °C
Drep3rev	AAATTAAGAACCTCAAACATATAATAAAC	
Drep4for	TTATGATATGTCTATTGATTATTG	51.5 °C
Drep4rev	CCTATATCTTTTCAAAATATTATTC	
Drep5for	CTTGTGCTCAGAGATATTCTTATGA	60.4 °C
Drep5rev	AAAAAATATACAACATCGATGTAG	
Drep6for	TAAAAGTAGCGTAAGTGATTG	60.4 °C
Drep6rev	AAAAAATAATAACCACAAATCTCCTGA	
