# Supplementary material for: The Mitochondrial Genomes of the Zoonotic Canine Filarial Parasites Dirofilaria (Nochtiella) repens and Candidatus Dirofilaria (Nochtiella) Honkongensis Provide Evidence for Presence of Cryptic Species
Source: PLoS Negl Trop Dis. 2016 Oct 11;10(10):e0005028. doi: 10.1371/journal.pntd.0005028 (PMC5058507; doi:10.1371/journal.pntd.0005028)
Supplement: S2 Table — (PDF) [file pntd.0005028.s002.pdf]

S2 Table. Mapping and assembly parameters for CLC Genomics Workbench 7.0.4

	Mapping parameters	Assembly parameters
Masking mode	no masking	Map reads back to contigs (slow)
Update contigs		Yes
Automatic bubble size		Yes
Minimum contig length		1000
Automatic word size		Yes
Perform scaffolding		No
Auto-detect paired distances		Yes
Mismatch cost	2	2
Insertion cost	3	3
Deletion cost	3	3
Length fraction	0.85	0.85
Similarity fraction	0.85	0.85
Global alignment	No	
Auto-detect paired distances	Yes	
Non-specific match handling	map randomly Output mode = create stand-alone read mapping	
Create list of un-mapped reads		No
