# Supplementary material for: The Mitochondrial Genomes of the Zoonotic Canine Filarial Parasites Dirofilaria (Nochtiella) repens and Candidatus Dirofilaria (Nochtiella) Honkongensis Provide Evidence for Presence of Cryptic Species
Source: PLoS Negl Trop Dis. 2016 Oct 11;10(10):e0005028. doi: 10.1371/journal.pntd.0005028 (PMC5058507; doi:10.1371/journal.pntd.0005028)
Supplement: S3 Table — (PDF) [file pntd.0005028.s003.pdf]

S3 Table. MiSeq read information

		Sample ID		
		**AW (dog, Italy)**	**S8 (human, Croatia)**	**S6 (human, India)**
**Raw Reads**	Number	2,842,040	2,290,512	5,751,974
	Nucleotides	467,156,581	257,472,454	810,753,057
	Phred Score >=30	78.72%	93.44%	94.27%
	Average length	164	112	141
**Mapped Reads**	Number	27,678	20,441	307,416
	Nucleotides	3,059,234	2,035,914	48,487,366
	average length	110	100	158
	Coverage	224	147	3545
