# Supplementary material for: The Mitochondrial Genomes of the Zoonotic Canine Filarial Parasites Dirofilaria (Nochtiella) repens and Candidatus Dirofilaria (Nochtiella) Honkongensis Provide Evidence for Presence of Cryptic Species
Source: PLoS Negl Trop Dis. 2016 Oct 11;10(10):e0005028. doi: 10.1371/journal.pntd.0005028 (PMC5058507; doi:10.1371/journal.pntd.0005028)
Supplement: S4 Table — (PDF) [file pntd.0005028.s004.pdf]

S4 Table. Nucleotide identity (%) in pairwise comparisons between complete mitochondrial genomes of members of the genera *Dirofilaria* and *Onchocerca*.

		D. repens				*C.* D. hong-kongensis	*D. immitis*	*O. volvulus*	*O. ochengi*	*O. flexuosa*
		Accession no.								
Species	Accession no.	KX265047	KX265048	KX265049	KR071802	KX265050	AJ537512	AF015193	mOo.2.0.fna[a]	HQ214004
D. repens	KX265047		99	99	99	94	87	86	86	86
	KX265048	99		99	99	94	87	86	86	86
	KX265049	99	99		99	94	87	86	86	86
	KR071802	99	99	99		94	87	86	86	86
C. D. hongkongensis	KX265050	94	94	94	94		85	85	85	85
D. immitis	AJ537512	87	87	87	87	85		84	85	85
O. volvulus	AF015193	86	86	86	86	85	84		97	89
O. ochengi	mOo.2.0.fna[a]	86	86	86	86	85	85	97		89
O. flexuosa	HQ214004	86	86	86	86	85	84	89	89	

Comparisons within *D. repens* are labelled green, while most inter-species comparisons are labeled red. The comparisons within sibling species pairs, i.e. *D. repens* vs. *C.* D. hongkongensis and *O. volvulus* vs. *O. ochengi*, are marked in yellow.

[a]Internal designation and not a GenBank accession number.
