# Supplementary material for: The Mitochondrial Genomes of the Zoonotic Canine Filarial Parasites Dirofilaria (Nochtiella) repens and Candidatus Dirofilaria (Nochtiella) Honkongensis Provide Evidence for Presence of Cryptic Species
Source: PLoS Negl Trop Dis. 2016 Oct 11;10(10):e0005028. doi: 10.1371/journal.pntd.0005028 (PMC5058507; doi:10.1371/journal.pntd.0005028)
Supplement: S5 Table — (PDF) [file pntd.0005028.s005.pdf]

S5 Table. Nucleotide identity (%) and amino acid identity (similarity) (%) in pairwise comparisons between protein and rRNA coding genes in the mitochondrial genomes of *Dirofilaria repens*, *Candidatus* Dirofilaria hongkongensis (*C.* D. hong.) and *Dirofilaria immitis*.

Gene	*D. repens*/*C.* D. hong.		*D. repens*/*D. immitis*		*C.* D. hong./*D. immitis*	
	cDNA	Protein	cDNA	Protein	cDNA	Protein
nduo-2	92.5	97.5 (98.2)	84.3	79.2 (89.8)	83.2	80.6 (90.1)
nduo-4	93.6	94.6 (98.8)	88.3	86.1 (93.9)	88.0	87.8 (94.1)
ctc-1	95.4	98.2 (99.5)	90.8	95.1 (97.3)	90.0	94.7 (97.4)
nduo-6	90.7	85.5 (90.1)	84.5	79.6 (83.7)	87.2	82.0 (90.0)
ctb-1	94.9	96.1 (99.2)	88.4	87.8 (92.5)	87.5	85.6 (91.7)
ctc-3	93.1	94.2 (98.1)	88.5	83.7 (91.1)	88.2	86.4 (91.1)
ndfl-4	95.5	96.3 (97.5)	90.1	86.3 (91.3)	88.5	82.5 (90)
rrnS	97.5		91.7		92.0	
nduo-1	95.1	98.7 (99.7)	88.2	86.3 (92.0)	88.7	87.0 (92.3)
atp-6	94.4	94.2 (96.9)	84.5	77.0 (78.0)	84.9	76.4 (83.8)
ctc-2	94.7	96.1 (98.3)	89.8	90.9 (97.4)	89.4	90.9 (96.6)
rrnL	96.6		87.1		86.9	
nduo-3	96.1	100	84.9	76.1 (82.9)	85.8	77.8 (83.8)
nduo-5	95.2	96.6 (97.7)	88.2	84.0 (91.3)	87.1	83.0 (91.1)
