# Supplementary material for: The Mitochondrial Genomes of the Zoonotic Canine Filarial Parasites Dirofilaria (Nochtiella) repens and Candidatus Dirofilaria (Nochtiella) Honkongensis Provide Evidence for Presence of Cryptic Species
Source: PLoS Negl Trop Dis. 2016 Oct 11;10(10):e0005028. doi: 10.1371/journal.pntd.0005028 (PMC5058507; doi:10.1371/journal.pntd.0005028)
Supplement: S1 Fig — For every amino acid as well as for Start and Stop codons the absolute number (A) and the relative synonymous codon usage (i.e. number of codons divided by the frequency expected if all synonymous codons would occur with equal frequencies) (B) are shown as stacked bar plots. For every amino acid, the left bar shows values for D. repens and the right bar for D. immitis. The codons encoding the different amino acids are numbered and color-coded as indicated in (C). (PDF) [file pntd.0005028.s006.pdf]

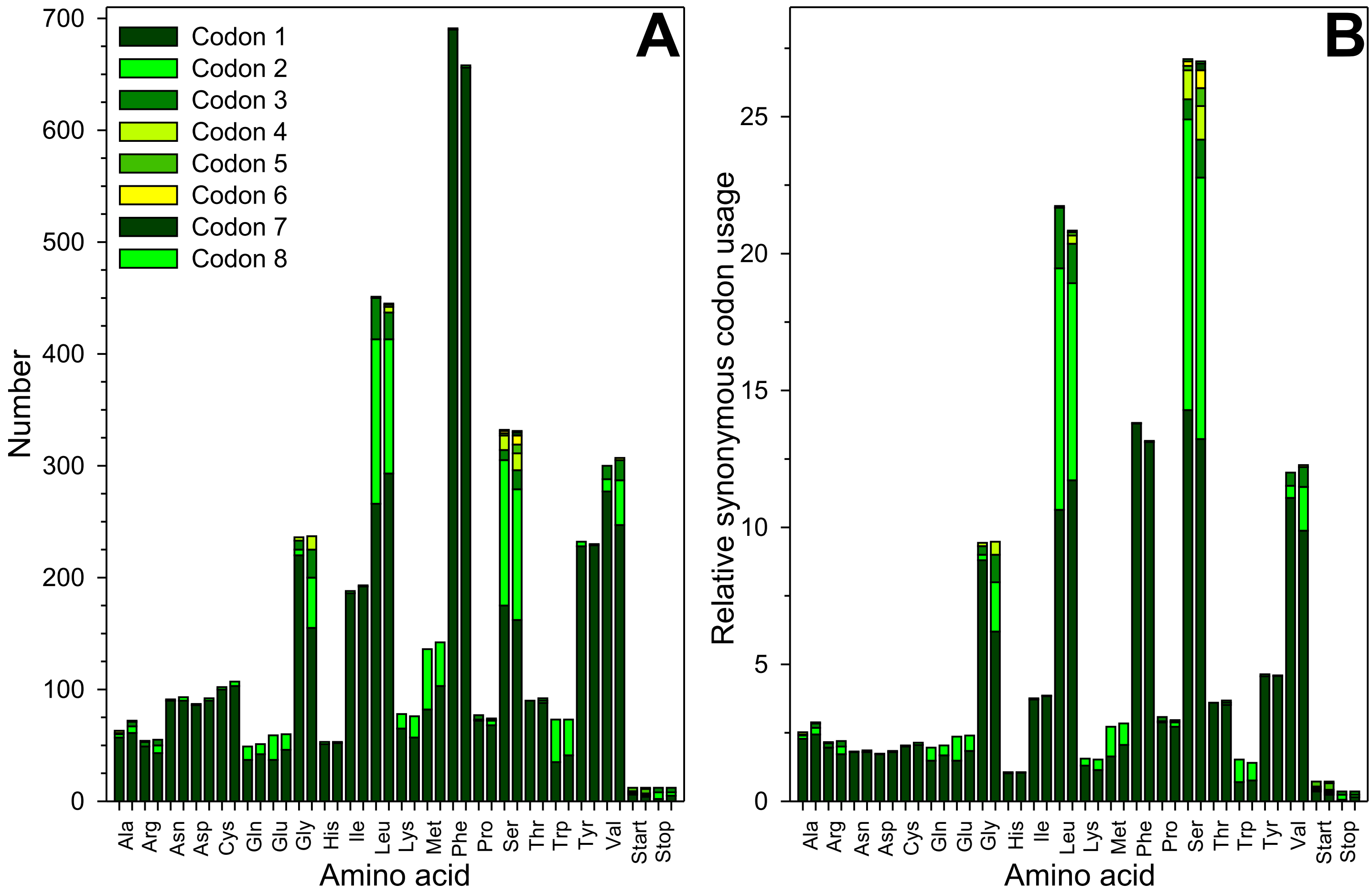

	Codon							
Amino acid	1	2	3	4	5	6	7	8
Ala	GCT	GCG	GCC	GCA				
Arg	CGT	CGG	CGA	CGC				
Asn	AAT	AAC						
Asp	TGT	TGC						
Cys	TGT	TGC						
Gln	CAG	CAA						
Glu	GAG	GAA						
Gly	GGT	GGG	GGA	GGC				
His	CAT	CAC						
Ile	ATT	ATC						
Leu	TTG	TTA	CTT	CTG	CTA	CTC		
Lys	AAG	AAA						
Met	ATG	ATA						
Phe	TTT	TTC						
Pro	CCT	CCG	CCA	CCC				
Ser	TCT	AGT	AGG	AGA	TCA	TCG	AGC	TCC
Thr	ACT	ACA	ACG	ACC				
Trp	TGG	TGA						
Tyr	TAT	TAC						
Val	GTT	GTG	GTA	GTC				
Start	ATT	TAT	GTA	CTT	TTG	GTT		
Stop	TAG	TAA	T					
