# Supplementary figures and images for: The Mitochondrial Genomes of the Zoonotic Canine Filarial Parasites Dirofilaria (Nochtiella) repens and Candidatus Dirofilaria (Nochtiella) Honkongensis Provide Evidence for Presence of Cryptic Species
Source: PLoS Negl Trop Dis. 2016 Oct 11;10(10):e0005028. doi: 10.1371/journal.pntd.0005028 (PMC5058507; doi:10.1371/journal.pntd.0005028)

### ***Dirofilaria repens***

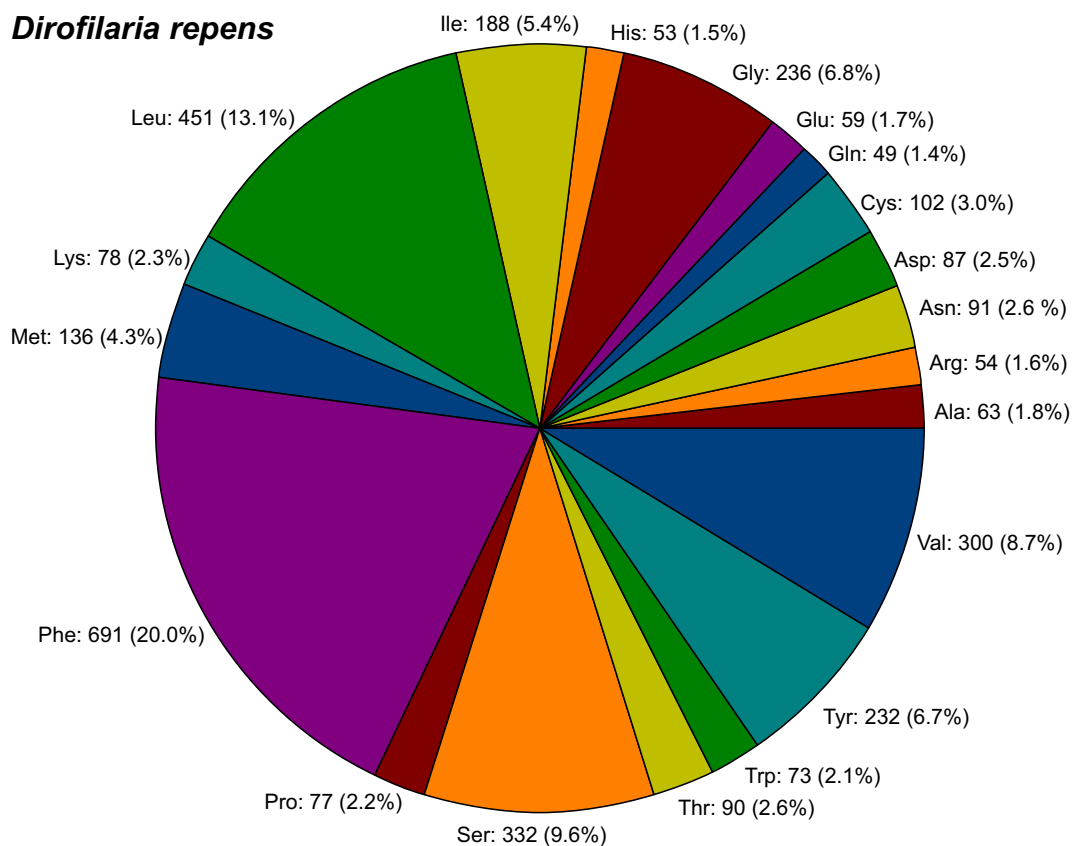

### ***Dirofilaria immitis***

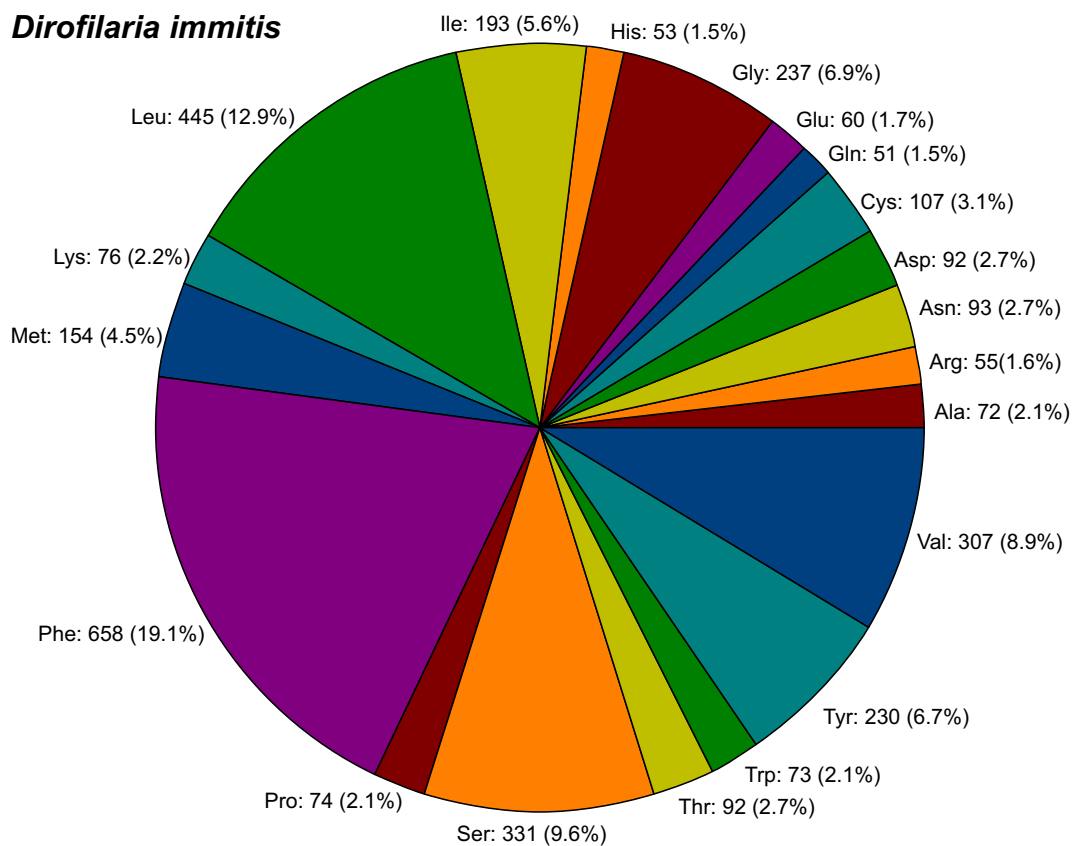

Supplement: S2 Fig — Absolute numbers of amino acids are given with frequencies in the genome in brackets. (PDF) [file pntd.0005028.s007.pdf]
