# Supplementary material for: The Mitochondrial Genomes of the Zoonotic Canine Filarial Parasites Dirofilaria (Nochtiella) repens and Candidatus Dirofilaria (Nochtiella) Honkongensis Provide Evidence for Presence of Cryptic Species
Source: PLoS Negl Trop Dis. 2016 Oct 11;10(10):e0005028. doi: 10.1371/journal.pntd.0005028 (PMC5058507; doi:10.1371/journal.pntd.0005028)
Supplement: S1 Supporting Information — (PDF) [file pntd.0005028.s008.pdf]

S1 Supporting Information. Assembly details for genomes from Illumina reads

Sample_ S6 (GenBank assecion number KX265050:

Reads were first mapped to the *Dirofilaria immitis* mitochondrial genome sequence (GenBank accession number AJ537512) using CLC Genomics Workbench 7.0.4 to sort out mitochondrial reads from contamination with chromosomal and bacterial reads (default parameters in these analyses as given in S2 Table except for length fraction = 0,87 and similarity fraction = 0.92). With the names of these reads, partner reads were extracted using the command-line-script in S2 Supporting information.

These reads were then assembled *de novo* (length fraction = 0.85, similarity fraction = 0.92). Comparison revealed that at about 6700-6800 bp, a 150 bp sequence with tRNA-Ala was deemed missing. This sequence was inserted from reference KX265047 and reads were mapped to confirm this to this consensus (Length fraction = 0,85, Similarity fraction = 0,87). The reads-based consensus was extracted as mitochondrion sequence.

Sample_ AW (GenBank assecion number KX265048:

Reads were assembled de novo (default settings except for length fraction = 0,85, similarity fraction = 0,92). A contig resembling worm mitochondrion was identified by Blasting the worm S6 mitochondrion against the assembly. The consensus sequence was cut, reordered to start at the same position as Wurm S6 and concatenated, with the consensus confirmed via read mapping (Length fraction = 0,87, Similarity fraction = 0,92).

Sample_ S8 (GenBank assecion number KX265049:

Reads were assembled de novo (default settings except for length fraction = 0,85, similarity fraction = 0,92) and the contig resembling worm mitochondrion was identified by Blast as above. Sequence was cut, reordered to start at the same positionas worm S6 and concatenated. A 362 bp sequence of Adult Wurm S6 was used to closed circular structure, and this was confirmed by mapping reads again to resulting consensus (length fraction = 0,87 similarity fraction = 0,92).
