# Supplementary material for: The Mitochondrial Genomes of the Zoonotic Canine Filarial Parasites Dirofilaria (Nochtiella) repens and Candidatus Dirofilaria (Nochtiella) Honkongensis Provide Evidence for Presence of Cryptic Species
Source: PLoS Negl Trop Dis. 2016 Oct 11;10(10):e0005028. doi: 10.1371/journal.pntd.0005028 (PMC5058507; doi:10.1371/journal.pntd.0005028)
Supplement: S3 Supporting Information — (PDF) [file pntd.0005028.s010.pdf]

S3 Supporting Information. Description of nucleotide composition content and nucleotide skews in the coding strand, non-coding regions and use of different stop and start codons

The AT content of both genomes is very high (76%). On the coding strand, T is by far the most frequently found base (56.9% *D. repens*/56.6% *C.* D. hongkongensis) followed by A (19.1%/19.7%), G (17.4%/16.9%) and C (6.5%/6.8%). This is quite similar to the *D. immitis* mitochondrial genome with an overall AT content of 74.2% (T 54.9%, A 19.3%, G 19.3% and C 6.6%). The AT skews of the coding strand of the *D. repens* and *C.* D. hongkongensis mitochondrial genomes were calculated to be -0.496 and -0.483, respectively (*D. immitis* -0.481). The GC skews were 0.453 and 0.447 (*D. immitis* 0.492). While the latter is in the same range as observed in many other filarial genomes, the AT skew for *D. repens* has the lowest value observed in any of the mitochondrial genomes of spirurid mitochondrial genomes sequenced up to now [76-78].

As observed in many, particularly invertebrate mitochondrial genomes, the intergenic space is reduced to a minimum and actually some genes even overlap. In spirurid nematodes, the AT-rich control region (CR) is the only longer non-coding region (284 bp and 274 bp in *D. repens* and *C.* D. hongkongensis). This is at the lower end of the range observed in spirurid nematodes with the shortest reported CR in *W. bancrofti* (264-267 bp) and the longest in *S. digitata* (506 bp). However, it is in marked contrast to a very long CR in some plant parasitic nematodes such as *Bursaphelenchus xylophilus* (1,650 bp), *Pratylenchus vulnus* (6847 bp) and *Meloidogyne graminicola* (2,031 and 5,063 bp) [79, 80], which is even split in two parts for the latter. For *D. repens*, the size of the remaining intergenic regions range between -22 and +47 bp (median 1 bp, mean 3.2 bp ± 9.9 bp) with negative values indicating overlap of genes. Similarly, spacer size in the mitochondrial genome of *C.* D. hongkongensis is between -22 and +49 (median 1, mean 3.5 bp ± 10.1 bp). While most spacers and overlaps are very small (-4 - +7 bp), there is considerable overlap between *nduo-1* and *trnF* (22 bp in both genomes). The largest spacers are found between *trnW* and *nduo-6* (47 bp and 49 bp), *trnI* and *trnG* (21 bp and 9 bp), *nduo-5* and *trnF* (12 bp and 9 bp), *nduo-2* and *trnT* (9 bp and 13 bp), *ctc-1* and *trnW* (9 bp in both genomes) and *atp-6* and *trnI* (8 bp and 7 bp) (Table 1). There is a tendency of longer intergenic spacers in *D. immitis* (median 1 bp, medium 4.5 bp ± 12.3), which is in accordance with the larger genome size of this species. However, this difference was not statistically significant (p = 0.33 in a paired One-Way-ANOVA).

None of the protein coding genes in the *D. repens* and *C.* D. hongkongensis mitochondrial genome starts with an ATG start codon (Table 1) but all start codons are conserved between these two species. In comparison to the initiation codons, the stop codons were less conserved between both species. In *D. repens*, the predominant stop codon was TAA (six genes), TAG was found in two genes. In four genes, the stop codon is represented by a single T, which is completed to TAA during polyadenylation of the transcript. In *C.* D. hongkongensis, TAA terminated only three open reading frames while TAG was found in five. Three open reading frames ended with T and one with TA. The degree of conservation of stop codons between *D. immitis* and *D. repens* is also only moderate (S1 Fig).
